# The proton therapy research beamline at the Christie NHS foundation trust

**DOI:** 10.1088/2057-1976/addbe8

**Published:** 2025-07-25

**Authors:** Nicholas T Henthorn, John-William Warmenhoven, Samuel P Ingram, Samuel P Manger, Michael J Merchant, Hywel Owen, Ranald I Mackay, Karen J Kirkby, Michael J Taylor

**Affiliations:** 1 Division of Cancer Sciences, Faculty of Biology, Medicine and Health, The University of Manchester, Manchester, United Kingdom; 2 Manchester Academic Health Science Centre, The Christie NHS Foundation Trust, Manchester, United Kingdom; 3 Christie Medical Physics and Engineering, The Christie NHS Foundation Trust, Manchester, United Kingdom; 4 The Cockcroft Institute, Warrington, United Kingdom; 5 ASTeC, UKRI-STFC Daresbury Laboratory, Warrington, United Kingdom

**Keywords:** proton therapy, medical physics, radiobiology, irradiation facility

## Abstract

Proton therapy is a relatively new modality for cancer treatment and has several open research questions, particularly in the biological realm. Due to large infrastructure costs the modality is reserved for specialist treatment, limiting the patient outcome dataset. This requires supplementation with fundamental research through *in vitro* and *in vivo* systems. Similarly, the safety and potential benefits of new treatments, such as FLASH, should be demonstrated in lab environments prior to clinical translation. Greater access to clinically relevant research platforms is required. This work presents the capabilities of the Manchester proton therapy research facility for experimentalists’ assessment to meet their research goals. Details of the research beamline geometry are presented, along with workflows for *in vitro* sample irradiation within an automated sample handling environmental chamber. Absolute dose and dose depth of the proton research beamline was measured. The dose calibration across a range of energies and dose rates is presented and fits are mathematically described. Methods to convert measured, or planned, dose to sample dose are presented including for biological studies investigating end of proton range effects. Elements of the beam optics, impacting on spot size and therefore field homogeneity, were measured for sample irradiation and beam model development. A Monte Carlo beam model was established to predict physically difficult measurements and is compared to measurements throughout. Achievable dose rates for FLASH are presented alongside absolute dosimetric accuracy. There was a focus on radiobiological research in establishing the beamline. Special care was taken to develop high-throughput repeatable *in vitro* irradiation workflows, with an adjacent radiobiological lab for immediate processing. This will lead to a reduction in experimental uncertainties seen in the literature with demonstrated accurate dosimetry, tight environmental control, and a high degree of versatility. The infrastructure presented in this work is a unique facility in the UK.

## Introduction

High-energy protons have been used for radiotherapy since the 1950s (Giap and Giap 2012), and domestically within the UK since 2018 with two National Health Service (NHS) centres (The Christie NHS Foundation trust and University College London Hospitals). Ocular treatments were available since 1989 (Kacperek 2009). The justification of these NHS centres is owed, in part, to the favourable physical dose distribution reducing total integral dose (Burnet *et al* 2020). Utilising the proton Bragg peak for tumour treatment can minimise, in some cases, the low dose bath to surrounding healthy tissue seen in many equivalent x-ray plans (Lomax *et al* 1999, Baumann *et al* 2016). However, this advantage comes at a cost of an increased sensitivity to uncertainties (Lowe *et al* 2020). The potential dose sparing can minimise normal tissue complication and the risk of secondary malignancies, shown for gastrointestinal (Badiyan *et al* 2018), breast (Dasu *et al* 2018), and malignant mediastinal lymphoma (König *et al* 2020). The latter forms the basis of the NHS indication list centred around paediatric and teenage & young adult patients (NHS England Specialised Services Clinical Reference Group for Radiotherapy 2020).

Despite the longevity of proton therapy, only ∼60 years younger than x-ray therapy, and relative success in patient treatment (Makishima *et al* 2015, Baumann *et al* 2020) and assumed benefit from clinical stakeholders (Burnet *et al* 2022) there remains a number of active research questions aimed at refining treatment (Lomax 2018) and access (Yan *et al* 2023). Several open questions still require further experimental research and evidence-based medicine (Chen *et al* 2023), since x-rays are the predominant radiotherapy tool with protons used in <1% of treatments (Mohan 2022). Other questions can be addressed through more fundamental *in vitro* and *in vivo* research offering insights and forming the basis of subsequent clinical trials. Most proton radiobiology research questions are focussed on translating existing experience with x-ray therapy, for example, optimised dose prescriptions for tumour control probability and normal tissue complication probability (Langendijk *et al* 2021), fractionation schedules (Kim *et al* 2019), the differential role of the immune system (Du *et al* 2021), etc Similarly, novel radiotherapy research topics have emerged that may lend themselves more favourably to protons over x-rays, e.g. ultra-high dose rate through the FLASH effect (Tang *et al* 2024) and spatially fractionated therapy (Prezado 2022, Sheikh *et al* 2024).

A large body of experimental data exists for the biological effect of protons. One key topic here is the Relative Biological Effectiveness (RBE), clinically implemented with a fixed value of 1.1; stating that protons are 10% more effective at cell kill than x-rays for the same given dose (Paganetti *et al* 2002). There is little clinical evidence to contradict this (Underwood *et al* 2022), with the existing evidence characterised as imaging changes in follow up MR (Peeler *et al* 2016, Bertolet *et al* 2022) or CT (Underwood *et al* 2018). However, several *in vitro* results show a variable RBE depending on dose, tissue type, and linear energy transfer (LET) (Friedrich *et al* 2013, Paganetti 2014). The *in vitro* evidence shows significant variation between datasets demonstrating a lack of experimental repeatability. Several variable RBE models have been developed that reproduce the general trends of this experimental data based on phenomenological modelling of the linear-quadratic formula (McNamara *et al* 2015, Rørvik *et al* 2018) or based on physical characteristics of the delivery (McMahon *et al* 2018, Henthorn *et al* 2023). However, none of these models are formally used as part of treatment planning in proton therapy, though such models may be used to guide decision making (Giovannini *et al* 2016, Mein *et al* 2022). The lack of clinical uptake is due, in part, to low model confidence arising from the variable experimental data (Mohan *et al* 2017, Friedrich *et al* 2021). This problem of experimental RBE data is seen in other radiobiological endpoints, such as micronuclei formation and chromosome aberrations (Heaven *et al* 2022, Endesfelder *et al* 2023). Radiobiological variability can be slightly controlled by relative measurements within the same lab (Nuryadi *et al* 2018), alluding to the impact of experimental protocols (including irradiation). For translational application of experimental data, the measurements must be made in a highly repeatable and high throughput manner with strict adherence to protocols and complete descriptions of experimental procedures and irradiation details (Durante *et al* 2019, Draeger *et al* 2020).

Addressing these uncertainties and improving current clinical deliveries requires more research capacity and greater access to research facilities (Durante 2019, Henthorn *et al* 2020). Recognising the need for research, the NHS procurement made provisions for a proton therapy research platform to operate alongside clinical service. Part of this was the allocation of a research room, occupying the fourth gantry room at the Christie NHS Foundation Trust. This room, operated by the University of Manchester in collaboration with the Christie, has a fixed horizontal beamline coupled to the Varian beam transport system and ProBeam cyclotron, further information on the ProBeam system can be found in Langner *et al* (Langner *et al* 2017). Research beamtime is made available outside of clinical hours, with a current maximum of 44 h per month. The facility is primarily used for medical physics and *in vitro* radiobiological experiments, with a versatile space near the nozzle where experimental ‘endstations’ can easily be switched. Similar efforts to widen research access to protons have been made at other high-energy clinical facilities (Tommasino *et al* 2019, Yang *et al* 2023), low-energy clinical facilities (Hofverberg *et al* 2022), and dedicated research facilities (Baratto-Roldán *et al* 2020, Vilches-Freixas *et al* 2020). A summary of proton therapy research beamline capabilities across Europe has previously been summarised (Henthorn *et al* 2020).

This work presents the physical characteristics of the research beam, including dose accuracy, dose rates, LET, spot sizes, and field homogeneity as well as biological capabilities, including experimental endstations and laboratory capacity. The physical characteristics were specified at design to match neighbouring clinical gantries. Throughout, measured results are presented alongside simulations from the TOPAS beam model. This model is used to predict values at immeasurable positions, and is used, for example, to generate correction factors between measured dose and sample dose. The TOPAS beam model is routinely used to plan and optimise experiments such as spread-out Bragg peak (SOBP) deliveries. In the work we present workflows employing these physical characteristics for radiobiological studies. With this information, potential users of the resource can gauge applicability of the facility to address their scientific question.

## Methods

### The research beamline

The research beamline occupies the fourth gantry room at the Christie proton beam therapy centre. The beamline is coupled to the Varian ProBeam cyclotron by the same beam transport system that supplies the clinical gantries. It is isolated by a 130 mm physical airgap with vacuums protected either side by 125 μm thick Kapton windows (DKW). Steerer magnets are used to centre the beam vertically and horizontally, and a quadrupole triplet is used to focus the beam to a plane 6415 mm downstream of the final quadrupole (focal plane). The steerers have a 60 mm aperture and an effective magnetic length of 132 mm. The quadrupoles have a bore diameter of 78 mm and a magnetic length of 273 mm. The quadrupoles are operated in the linear mode (<100 A), where increasing power linearly increases magnetic field strength. All magnets were manufactured by Sigma Phi and driven by Sigma Phi power supplies, capable of providing up to 200 A for quadrupoles and 22 A for steerers. The beamline has two actuated beam profile monitors (BPM) either side of the quadrupole triplet, supplied by Pyramid technical consultants (Pyramid, USA), composed of 16 × 16 strips with a pitch of 2.5 mm. Vacuum is maintained at ∼1E-6 mbar by two Pfeiffer HiPace 300 turbo pumps each backed by a Pfeiffer ACP15 roughing pump. The pumps are evenly spaced along the beamline and separated by a remotely operated gate valve, which can be used to isolate each half of the research vacuum system. The beamline terminates with a Varian engineering scanning nozzle, containing standard Varian scanning magnets capable of scanning the beam over a maximum field size of 400 × 300 mm at the focal plane, matching the clinical capabilities. The scanning magnets are driven by a Copley model C78X pulse-width modulator power amplifier system.

Online monitoring and control of beam delivery is achieved using the air-filled MSIC, coupled to an IC 101 electrometer (Pyramid, USA) and a CompactRIO field programmable gate array (FPGA) (National Instruments, USA). The MSIC, matching the clinical gantries, contains two integral planes and a set of horizontal and vertical strips. Only one of the integral planes is used in the research beamline. The IC 101 is used to bias the MSIC to −1000 V and integrate the collected charge. The charge is integrated on a 100 pF capacitor, as a voltage, using an integration time of 111 μs, resulting in a maximum collectable current of 5.6 μA from the MSIC. Resetting the capacitor takes an additional 49 μs, resulting in an overall 32.7% dead time for every sampling period, which is accounted for in the fitting process of dose per MU (equation (4)). During this dead time the FPGA is still active. A single pair of voltage readings is taken over the integration period and no averaging of multiple sampling periods is done. A gain is applied to the measured voltage before a monitor port on the electrometer reports it via a ±10 V signal. The gain is set to 5 for conventional dose rates and 1 for Ultra High Dose Rates (UHDR). This ±10 V signal is in turn integrated by the FPGA at a 25 kHz sampling rate and compared to a voltage target. Voltage targets are supplied by the user in terms of monitor units (MU), which are automatically converted by the beam control software to a voltage and adjusted for temperature and pressure. The control system allows the FPGA to terminate beam delivery once the required MUs have been recorded.

**Figure 1.**
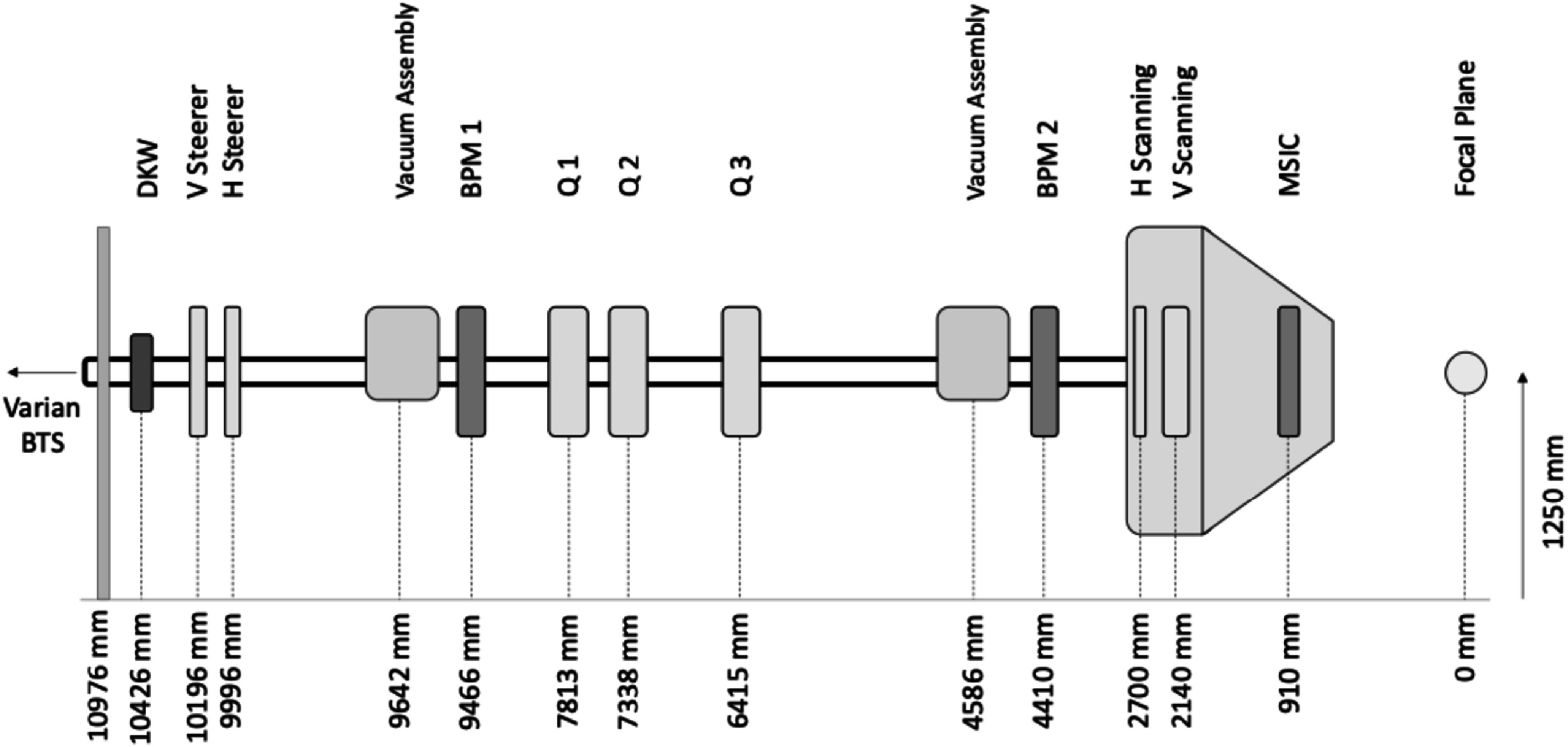
Schematic diagram of the proton research beamline, not to scale. Diagnostic elements are coloured red, including the multi-strip ionisation chamber (MSIC) for real-time beam monitoring and dosimetric control and two beam profile monitors (BPM) for beam optics measurement. Magnetic elements are coloured green, including vertical (V) and horizontal (H) scanning magnets, three quadrupoles (Q1–Q3) for beam focussing, and horizontal (H) and vertical (V) steerers for beam alignment. Vacuum systems are coloured blue. The dual Kapton window (DKW) is a 130 mm physical airgap separating the Varian beam transport system (BTS) from the research beamline.

The beam is focussed to a plane where a versatile space is provided for interchangeable experimental ‘endstations’. Alignment to the focal plane is facilitated by a set of Cemar MAXX-700 lasers, using one overhead and two horizontal lasers that are in turn aligned to a physical marker. The beamline is shown schematically in figure 1.

### Beam focussing and scanning

The steerer magnets are used to centre the beam through the quadrupoles along their bore axis. The magnetic field for the quadrupoles was initially estimated from beam optics simulations as part of the beamline design. Fine tuning of the quadrupole fields was carried out to minimise the Gaussian spot size at the focal plane across the energy range. Spot size measurements were made in air at the focal plane with a Lynx scintillating camera (IBA, Belgium) at 10 MeV intervals between 70–245 MeV. These were also measured proximal and distal to the focal plane (±200 mm in increments of 100 mm) for beam model development. Beam divergence with depth in air relative to the focal plane was fit using the Courant-Snyder formalism (Huang *et al* 2018), in both horizontal (H) and vertical (V) directions, shown in equation (1).

\begin{eqnarray*}\begin{array}{lll}{\sigma }_{H| V}^{2}\left(z\right) &amp; = &amp; {\sigma }_{H| V}^{2}\left(0\right)-2{\rho }_{H\left|V\right.}\left(0\right)\,{\sigma }_{H\left|V\right.}\\ &amp; &amp; \times \,\left(0\right)\,{\varnothing }_{H\left|V\right.}\left(0\right)\,z+{\varnothing }_{H\left|V\right.}^{2}\left(0\right)\,{z}^{2}\end{array}\end{eqnarray*}

Where, ${\sigma }_{H\left|V\right.}\left(z\right)$ is the spot sigma at depth z, with negative z being towards the nozzle. z = 0 indicates the parameter at the focal plane. ${\rho }_{H\left|V\right.}$ is the correlation coefficient (restricted between −0.99 and +0.99). ${\varnothing }_{H\left|V\right.}$ is the beam divergence. ${\rho }_{H\left|V\right.}\left(0\right)$ and ${\varnothing }_{H\left|V\right.}\left(0\right)$ are fit against the spot sizes measured with depth in air.

Optimal measured current settings for steerers and quadrupoles were fit through polynomials to interpolate between setpoints.

A spot map, a series of scanning magnet current settings, with well-spaced spot positions was delivered to the Lynx at the focal plane, in 10 MeV intervals between 70–245 MeV. Spot centroids were identified by fitting a 2D Gaussian profile. Position change, in mm, was measured as a function of change in scanning magnet current supply, in A. These measured values of mm/A were fit as a function of proton energy, E, with equation (2), using fitting parameters a and b.

\begin{eqnarray*}\displaystyle \frac{mm}{A}=a{E}^{b}\end{eqnarray*}

A 2 A tolerance on scanning magnet current supplies is used, corresponding to a positional displacement of 2.28 and 1.58 mm in the horizontal and vertical position respectively for the lowest energy (70 MeV). Spot position repeatability was measured by delivering 200 × 200 mm fields with 75 mm spot spacing to the Lynx at the focal plane, in 10 MeV intervals between 70–245 MeV, three times. Monitored feedback of the magnet current pauses beam delivery if the scanning magnet current power supplies go out of tolerance. The scanning magnet power tolerance was also reduced to 1.0 A and 0.5 A. These new tolerances effectively halve and quarter the spot positional uncertainty at the potential cost of longer delivery times. To assess any impact on delivery times, spot position repeatability fields were redelivered at the new tolerances.

Scan speed was determined through magnet power supply (Copley C78X, Analogic, USA) speed, with slew rate settings of 0.45 and 0.17 A/10 μs for the horizontal and vertical scanning magnets respectively. The scanning speed was validated with oscilloscope measurements on the cyclotron vertical deflector signal (the beam on/off signal) for a 245 MeV 100 × 100 mm field with 2.5 mm spot spacing. This was converted to metres per second through equation (2), giving 10.64 and 2.75 m s^−1^ in the horizontal and vertical directions respectively. For the horizontal direction the scan speed is in line with a similar Varian facility (Danish Centre for Particle Therapy) but is approximately 10 times slower in the vertical direction (Kanouta *et al* 2022). The scanning speeds are also in line with other dissimilar facilities, for example 19.3 and 5.9 m s^−1^ measured at the Mayo Clinic on the Hitachi ProBeat V5 system (Gelover *et al* 2019). Scanning speed as a function of energy is scaled from the 245 MeV value based on the ratio of relativistic momentum (figure S2). This gives a maximum scanning speed at the lowest energy, 70 MeV, of 20.78 and 5.37 m s^−1^ in the horizontal and vertical directions respectively.

Field dose homogeneity was measured with the Lynx at the focal plane in 10 MeV intervals between 70–245 MeV. 200 × 200 mm 0.5 Gy fields with 2.5 mm spot spacing to ensure sufficient spot overlap, with the central 50% of the field analysed for homogeneity index, HI, through equation (3) using pixel intensity, I.

\begin{eqnarray*}HI=100\times \displaystyle \frac{{I}_{\max }-{I}_{\min }}{{I}_{\max }+{I}_{\min }}\end{eqnarray*}

### Dose delivery calibration procedures

Dosimetric calibration of MU was carried out in a PTW MP3-M watertank (PTW, Freiburg), using a PTW Bragg peak chamber (Ref: TM34070-2.5) operating at +400 V and a PTW Roos chamber (Ref: TM34001) operating at +200 V both read out by a PTW UniDos E electrometer operating in medium range. The chambers were independently positioned at 20 mm water equivalent thickness (WET) and aligned to the focal plane. The Roos chamber has been independently calibrated on a neighbouring clinical gantry against the clinical Roos, which is in turn calibrated against the NPL primary standard (National Physical Laboratory, Teddington, UK) forming the calibration chain. The Bragg peak chamber is uncalibrated for absolute dose and only used for relative dosimetry.

The Bragg peak chamber was irradiated with five repeats of single spots across a range of energies (70–245 MeV), a range of cyclotron currents (10–800 nA), and a range of MU (50–1600 MU), collecting total charge from the entire spot. Following this, uniform 100 × 100 mm spot maps were created with a spot spacing of 2.5 mm for a 200 MeV at 200 nA cyclotron current beam, with the same range of MU per spot as for the single spot irradiations. These dimensions are large enough to ensure the Roos is in a homogeneous field, which was used to measure the absolute dose. The absolute field dose collected by the Roos was divided by the charge collected by the Bragg peak chamber for the single spots to generate a conversion between field dose and single spot charge. This value was used to convert all single spot Bragg peak chamber measurements into field doses. Lastly, uniform 100 × 100 mm spot maps were created with a spot spacing of 2.5 mm for the entire energy range, with a single cyclotron current selected for each energy and with 400 MU per spot. Absolute dose was measured with the Roos and used to validate the above conversions. This combined Bragg peak chamber and Roos data provides a dose per MU at 20 mm WET reference position over a range of energies, currents, and doses. This data is fitted with equation (4) detailed below, using a dose dependent component, *χ* (equation (5)), and a dose independent component, Δ (equation (6)). Equations (5) and (6) phenomenologically convert dose into MU, accounting for differences in LET at the MSIC and reference dosimetry position, electronic processing of the IC 101, and IC 101 deadtime. Initially *χ* and Δ are treated as free parameters in a first-pass fit. The value of *χ* is then plotted against energy and equation (5) is fit to this data. Having determined the value of *χ* as a function of energy, equation (4) is then re-fit with only Δ as a free parameter. A surface, equation (6), is then fit to the values of Δ from this pass, giving us the final form of equation (4) in terms of only energy and cyclotron current.

\begin{eqnarray*}\displaystyle \frac{D}{MU}=\displaystyle \frac{D}{\chi \left(E\right)D+{\mathrm{\Delta }}\left({I}_{C},E\right)}\end{eqnarray*}

\begin{eqnarray*}\chi \left(E\right)=-\exp \left({x}_{1}E+{x}_{2}\right)+{x}_{3}\end{eqnarray*}

\begin{eqnarray*}\begin{array}{lll}{\mathrm{\Delta }}\left({I}_{C},E\right) &amp; = &amp; \left(-\exp \left({d}_{1}E+{d}_{2}\right)+{d}_{3}\right)\\ &amp; &amp; \times \,{I}_{c}\left({t}_{1}{E}^{{t}_{2}}+{t}_{3}\exp \left({t}_{4}E\right)\right)\end{array}\end{eqnarray*}

Where D is the dose in Gy, E is the proton energy in MeV, and ${I}_{c}$ is the cyclotron current in nA. All other parameters are fit to measured data. ${I}_{c}\left({t}_{1}{E}^{{t}_{2}}+{t}_{3}\exp \left({t}_{4}E\right)\right)$ describes the transmission, converting cyclotron current into nozzle current, which was determined by measuring the current collected at the focal plane using a beam collector Faraday cup, BC-75 (Pyramid, USA), and comparing this to machine log files reporting cyclotron extraction current.

A set of dosimetry calibration fields (100 × 100 mm fields, 2.5 mm spot spacing, 400 MU/spot, 70–245 MeV) were repeated with dose collection by a PTW microDiamond chamber held in a modified 96-well plate (1.2 mm thick polystyrene) at the focal plane in air. This corresponding data set was used to generate a scaling factor to convert dose at reference position (20 mm WET) to ‘surface’ dose as a function of energy, where surface refers to a thin layer distal to the polystyrene. This scaling is used to determine MU required for dosing biological samples embedded in tissue culture plasticware, accounting for the dose buildup between the two depths. A further polynomial scaling is applied to correct from the 1 mm WET measurement depth of the microDiamond to the surface. The scaling as a function of energy, E, is given in equation (7) through five fitting parameters (a–f) describing correction from 20 mm to 1 mm WET, and a further three (g-i) describing correction from 1 mm WET to surface.

\begin{eqnarray*}\displaystyle \frac{{D}_{20\,mm}}{{D}_{Surface}}=\displaystyle \frac{a{E}^{b}+cE+d}{eE+f}\times \displaystyle \frac{1}{g{E}^{2}+hE+i}\end{eqnarray*}

Absolute dosimetry chambers, additional to the primary PTW Roos, were calibrated against the MSIC at 20 mm WET, including a secondary PTW Roos, three PTW microDiamonds (Ref: TM60019), a PTW Semi-Flex (Ref: TM31021), and a PTW Advanced Markus (Ref: TM34045).

### Beam energies

The energy selection system, just downstream of the cyclotron, degrades the 250 MeV protons to a minimum energy of 70 MeV. The research room is commissioned up to a maximum energy of 245 MeV. When experiments require energies below 70 MeV, further degradation is achieved with solid water (Sun Nuclear HE) attached to the nozzle. Through the combination of solid water and energy selection it’s possible to irradiate samples with energies between 0–245 MeV, extending the capabilities to study effects at various LETs.

Beam energy was verified through dose depth range measurements in a PTW MP3-M watertank (PTW, Freiburg), using a PTW Bragg peak chamber and a PTW thin window (Ref: TM34080-2.5). Relative dose measurements were made at 5 mm intervals, with increased resolution of 0.5 mm around the Bragg peak region. The range in water of the proximal 80% maximum dose was fit with a power law shown in equation (8) (Bortfeld 1997). Where E is the nominal proton energy in MeV, and a and p are parameters of the fit.

\begin{eqnarray*}{R}_{80}\left(mm\right)=a{E}^{p}\end{eqnarray*}

Validation of further energy degradation through solid water was confirmed with a PTW microDiamond in air at the focal plane. Under these conditions, our precalculated dosimetry calibrations (equation (4)) no longer apply. The microDiamond was irradiated with 100 × 100 mm fields at 2.5 mm spot spacing in the energy range of 70–80 MeV with 40 mm solid water attached to the nozzle. Cyclotron beam current was chosen to match nozzle currents across the energy range and in each case 100 MU per spot was used. The absolute dose measurements were normalised to the value of maximum dose across the energy range. This geometry was simulated in the Monte Carlo toolkit TOPAS v3.7, with the microDiamond modelled as a 1 mm buildup water plane and a distal 0.5 mm scoring water plane. The simulation was then rerun without the 1 mm buildup to measure dose and LET at the sample position. Dose-averaged and track-averaged LET were scored using the proton-LET TOPAS scorer. The ratio of simulated dose in the microDiamond to simulated dose at the sample position is used as a scaling factor for experimental delivery. Experimentally, the required MU for a dose of an energy degraded field is iteratively determined by measurement with the microDiamond and energy specific scaling factor.

### Dose rate

Typically, cyclotron current is selected to achieve a nominal 2 nA nozzle current, except at energies below 150 MeV where transmission limits nozzle current (figure 5(a)). To determine maximum achievable dose rates, the instantaneous dose rate was measured. A PTW microDiamond chamber was aligned to the focal plane in air and irradiated with a single spot of 245 MeV protons with a cyclotron current of 800 nA (giving maximum transmission). The beam was left on for up to 5 s with the microDiamond collecting charge read by the PTW UniDos E electrometer in high range mode, using a low resistor to avoid electrometer saturation. Cyclotron log files were used to determine cyclotron current during irradiation. Peak dose rate was determined by dividing dose collected by exposure time, accounting for the Gaussian spot profile and the 3.8 mm^2^ active area of the microDiamond. Dose rates at all other energies and cyclotron currents were inferred through scaling by measured transmission and spot size. This peak dose rate was used to calculate field dose rates according to the average dose rate definition (Folkerts *et al* 2020), though we account for the 0 to 100% dose rate.

Prior to UHDR sample irradiation the dose for a given MU is measured with a PTW microDiamond chamber on the Unidos E electrometer in high range. This is carried out at the beginning of each UHDR beam request to account for differences in cyclotron set-up, and the MU are tuned to achieve the required dose through automated scripting. Each UHDR sample has film attached as a secondary check of delivered dose. Full details of our UHDR irradiation process have been published previously (Aylward *et al* 2023).

### Beam model

The geometry shown in figure 1, from the horizontal scanning magnet onwards, was built in TOPAS v3.7 (Perl *et al* 2012, Faddegon *et al* 2020). The TOPAS physics list was composed of six modules (g4em-standard_opt4, g4h-phy_QGSP_BERT_HP, g4decay, g4ion-binarycascade, g4h-elastic_HP, and g4stopping). A beam model was developed by simulating dose depth curves and spot size measurements made at depth in air relative to the focal plane. The performance of the beam model is demonstrated throughout the results section alongside experimental measurements.

### The radiobiology endstation

**Figure 2.**
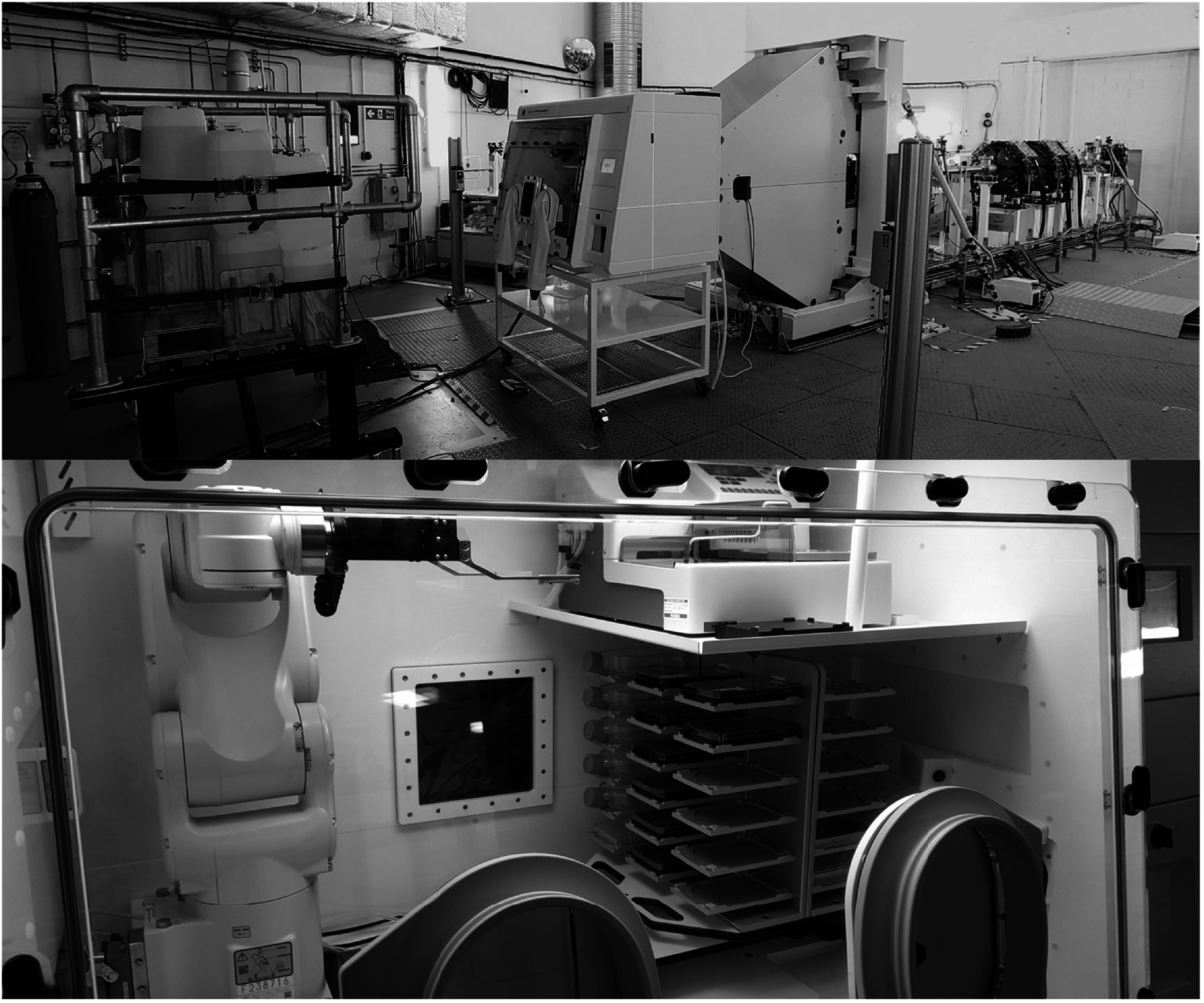
The radiobiology endstation provides high-throughput experiments in an environmentally controlled chamber. Biology samples, of the footprint of a 96-well plate, are loaded into the ‘hotels’. The 6-axis robot picks samples and holds them at the focal plane. The beam is scanned across the sample, entering the cabinet through two 125 um Kapton windows. 96-well plates can be fixed at specified timepoints with the WellWash Versa. The robot can hold a PTW microDiamond chamber at the focal plane for online dosimetry.

At the exit of the nozzle is a versatile space to position specific experimental ‘endstations’. The primary endstation for *in vitro* radiobiology experiments is a bespoke environmental cabinet (Don Whitley Scientific, UK), figure 2. This endstation is a modified Whitley H135 hypoxia cabinet with two 125 μm thick, 200 × 200 mm Kapton windows embedded in the rear of the cabinet, allowing the proton beam into the enclosed space. The cabinet contains a double-sided remotely rotatable ‘hotel’ for holding up to 36 biology samples (of the footprint of a 96-well plate or T75 flask). Other, smaller, samples can be used by 3D printing custom holders. A 6-axis LR Mate 200iD/4sc robotic arm (Fanuc, USA) selects the sample and holds it at the focal plane and the proton beam is scanned across the sample. This setup minimises intervention and downtime due to handling of activated samples. The cabinet can be environmentally controlled, with O2 levels between 0.1% to ambient, CO2 levels between 0 to 20%, temperature control between 7 °C to 45 °C, and humidity control between ambient and 100%. The cabinet also contains a WellWash Versa (Thermo Fisher Scientific, UK) that has been integrated with the robotic system for automated cell fixation in 96-well plates. Background dose to samples in the hotel during irradiation was measured with thermoluminescent diode badges positioned at various locations in the hotel, with the worst position showing 1.27 mGy per Gy delivered to a sample at the focal plane. Recently, the endstation has been expanded, doubling the internal working area, and providing space for an IX3 inverted fluorescent microscope (Evident, Germany) for early timepoint measurement of fluorescently tagged cells.

Typically, biological samples are irradiated in cell culture plasticware, without additional buildup material. Doses are planned (equations (4) and (7)) and verified with the PTW microDiamond held at the focal plane in the endstation. No correction is applied to scale the measured dose from the microDiamond to the cell layer adhered to the plasticware. This depth discrepancy, from the WET buildup of the microDiamond (1 mm), was investigated with the TOPAS beam model for typical plastic thicknesses (figure 6(b)).

Adjacent to the research beamline is a small biology lab designed as a staging area and for post irradiation treatment. This biolab is equipped with a tissue culture hood, incubators, hypoxic workstation, centrifuge, microscopes, etc Entire biology experiments can be performed in this lab, depending on their complexity.

### Quality assurance

Regular dosimetry checks are carried out at monthly and annual intervals and are reviewed at a monthly meeting. The nature of these quality assurance measurements are derived from the clinical service, based initially on guidance from the American Association of Physicists in Medicine (Arjomandy *et al* 2019). Bespoke dosimetry for some specific experiments can be verified prior to the experiment, particularly for experiments that require non-uniform fields. For example, to maximise dose rate in UHDR experiments it is usual to irradiate samples with a single horizontal line requiring precise positioning. In this case the beam centre relative to the sample is verified by scanning the beam across a PTW microDiamond, the required MU for a given dose is determined by delivering the horizontal line to the microDiamond, and the GafChromic^TM^ EBT3 film is used as a secondary check of delivered dose.

Monthly, our primary dosimetry chamber is checked for consistency by delivering known dose fields of 100 × 100 mm, with 2.5 mm spot spacing, across the energy range (70–240 MeV). All other chambers are calibrated against this Roos in the research beamline at the 20 mm water depth reference position, using a 100 × 100 mm field of 2.5 mm spot spacing with 224.5 MU per spot at 230 MeV. The spot size in air at the focal plane is checked monthly using the Lynx at 10 MeV intervals across the full energy range (70–245 MeV). The performance of the dosimetric calibration equation (4) is checked across the dose range at 70 MeV and at 245 MeV, with 245 MeV also checked across a range of nozzle currents. Laser alignment to the focal plane is checked with a physical device mounted to the concrete floor and realigned if lasers have drifted.

Quarterly, the Roos and Advanced Markus chambers undergo strontium checks as an independent integrity verification.

Annually, the integrated depth dose, spot size with depth in air, scanning performances, and field homogeneity are remeasured across the full energy range and the full dosimetry of equations (2), (4)–(8) are recommissioned.

## Results

### Beam focussing

**Figure 3.**
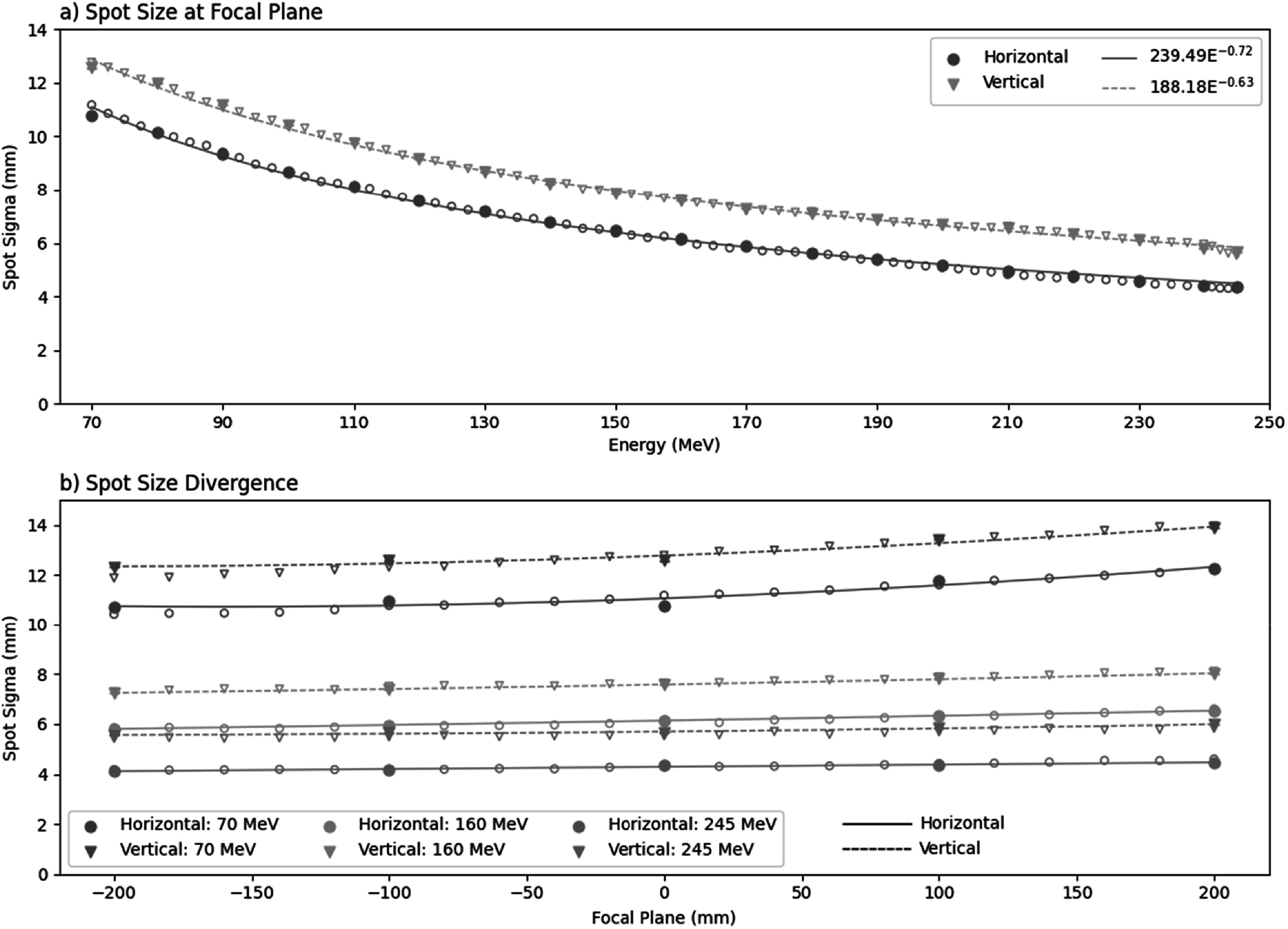
(a) The fitted Gaussian sigma of the spot at the focal plane in the horizontal and vertical direction across the energy range 70–245 MeV. (b) Spot size divergence relative to the focal plane for 70, 160, and 245 MeV, where negative is towards the nozzle. Spot sigma in the horizontal direction is shown as closed circles and solid lines whilst vertical is shown as open triangles and dashed lines. Line fit show Courant-Snyder beam optics model. Filled points show measurement, empty points show simulation, lines show fit to measurement. Both panels show uncertainty in fitted sigmas calculated from the covariance matrix (smaller than the marker size).

The spot size at the focal plane, given as 1*σ* of the Gaussian fit, (figure 3(a)) follows an energy dependent power law of $\sigma =239.494\times {E}^{-0.723}$ and $\sigma =188.177\times {E}^{-0.631}$ for the horizontal and vertical directions respectively. Lower energies have larger spot sigmas, 10.78 × 12.57 mm for 70 MeV, compared to higher energies, 4.35 × 5.65 mm for 245 MeV.

Spot size with depth in air relative to the focal plane (figure 3(b)) shows the expected behaviour of a slight increase, with the lowest energies showing greater divergence due to increased scattering in air. Spot sigma with depth relative to the focal plane was fit using the Courant-Snyder formalism (equation (1)), with fit parameters shown in supplementary table S1.

### Beam scanning

The millimetre shift as a function of scanning magnet A was fit to equation (2) with parameters ${\mathrm{a}}=10.944$ and ${\mathrm{b}}=-0.553$ for horizontal scanning and ${\mathrm{a}}=7.853$ and ${\mathrm{b}}=-0.542$ for vertical scanning (figure S1a). The measured spot position repeatability at 245 MeV, for a given scanning magnet power tolerance, is reported as the average standard deviation between repeat spot delivery positions (figure S1b) and shows <0.1 mm variation. This level of repeatability is similar to other facilities (Li *et al*) and is unlikely to impact field dose homogeneity (Arjunan *et al*). For all applied tolerances, the positional accuracy was below the theoretical upper limit specified by equation (2). The effect of scanning tolerance on delivery time is shown in figure S1c and, given error bars, shows no clear trend.

**Figure 4.**
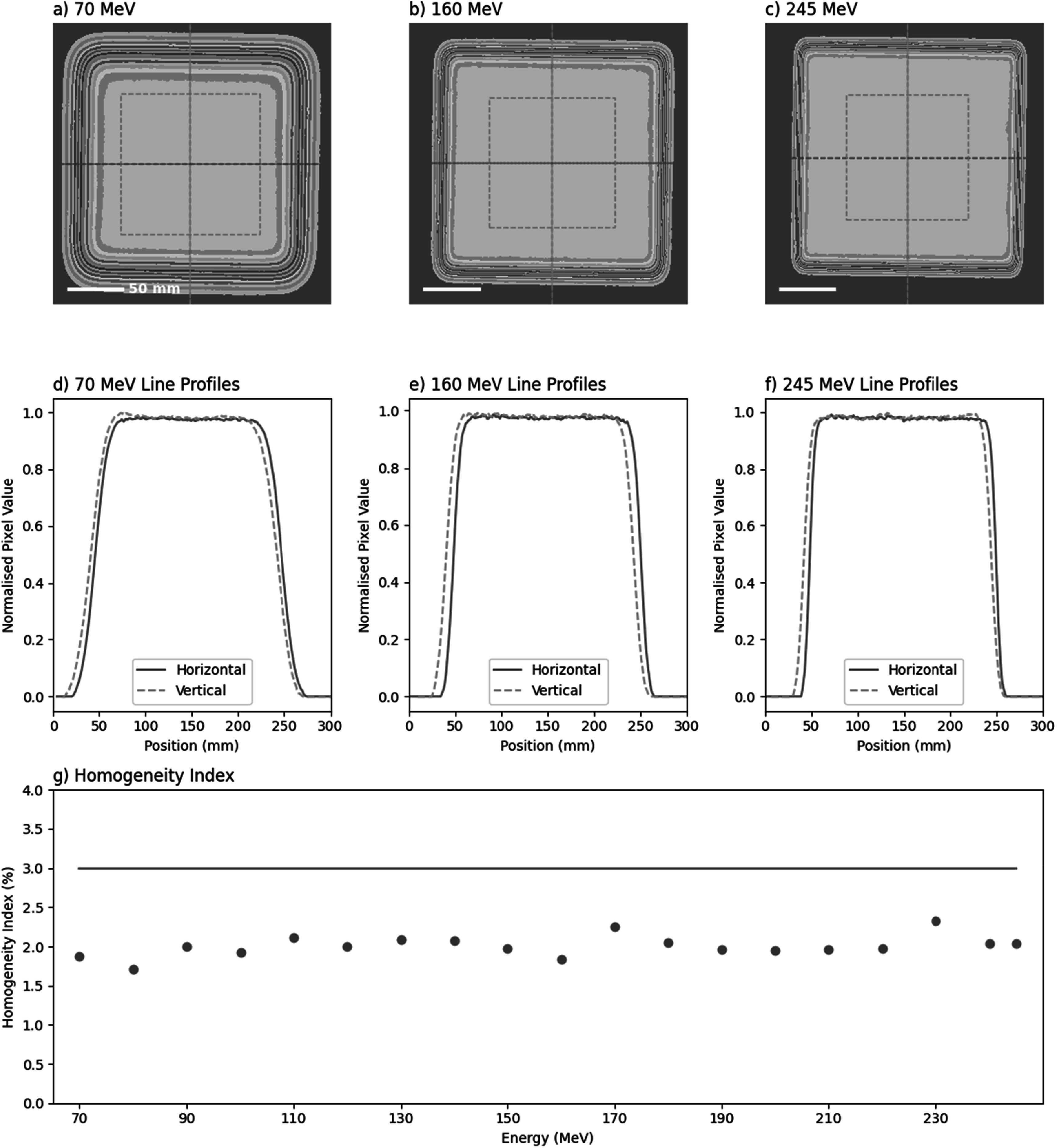
Homogeneity for uniform delivery of 200 × 200 mm fields with 2.5 mm spot spacing measured on the Lynx. Dose heatmaps for (a) 70 MeV, (b) 160 MeV, (c) 245 MeV with corresponding line profiles in the horizontal and vertical direction across the lynx shown in (d)–(f), denoted by dashed orange and blue lines on (a)–(c). (g) The central 50% area (shown in a)—c)) as the dashed pink box) was analysed for homogeneity across the 70–245 MeV energy range, red line shows a 3% tolerance.

The measured homogeneity index (equation (3)) was below a 3% threshold across the energy range showing no clear trend (figure 4(g)), with an average of 2.01 ± 0.14%. A 3% tolerance was chosen as a value to detect problems in steering and monitoring based on clinical experience. Figures 4(a)–(c) clearly show the increase in penumbra sharpness as spot size decreases with increasing energy. Horizontal and vertical line profiles show this sharpness quantitatively (figures 4(d)–(f)). A slight offset can be seen between vertical and horizontal measurements due to Lynx alignment.

### Dose delivery calibration procedures

**Figure 5.**
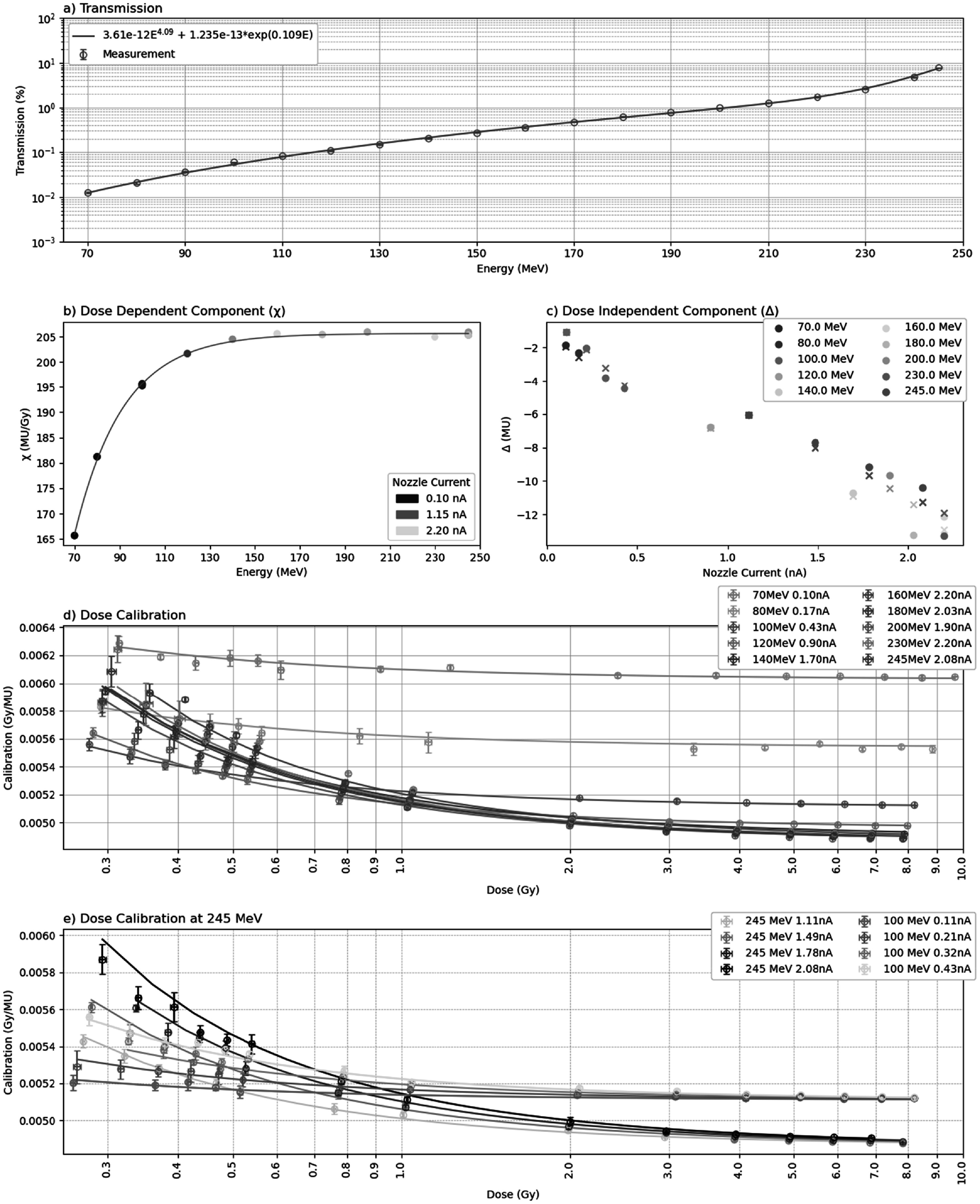
(a) Beam transmission from cyclotron measured at the focal plane with a BC-75 Faraday cup (Pyramid, USA), showing increasing transmission with energy. (b) The *χ* dose dependent component (equation (5)) as a function of energy showing an energy dependence, colours show nozzle current. (c) The Δ dose independent component (equation (6)) as a function of nozzle current, showing a decrease with increasing nozzle current and slight energy dependence, colours show energy. (d) The fit of equation (4) (lines) for dose per MU measured (points) with the PTW Bragg peak chamber and PTW Roos shown for a range of energies as a function of delivered dose. Data is shown once per energy, at the maximum delivered nozzle current. In all cases we see a low dose non-linearity and an energy/nozzle current dependent offset. (e) The performance of equation (4) for 245 and 100 MeV at varying nozzle currents. Energy largely determines the offset in Gy/MU at high doses whilst nozzle current determines the spread in calibration in the low dose non-linear region.

The beam transmission, from cyclotron to focal plane, as a function of energy is shown in figure 5(a). Transmission starts at 0.0125 ± 0.0004% at the lowest energy of 70 MeV and increases to a maximum of 7.8 ± 0.2% at the highest energy of 245 MeV. The rate of change in transmission with energy is nonconstant, with an increasing rate of change with increasing energy. It takes 50 MeV to increase transmission by a decade from 0.0125 ± 0.0004% at 70 MeV to 0.108 ± 0.002% at 120 MeV. Transmission steadily increases by another decade over the next 90 MeV, reaching 1.26 ± 0.03% for 210 MeV, and then rapidly rising to 7.8 ± 0.2% at 245 MeV.

The *χ* parameter (equation (5)), describing the dose dependent component of the dose per MU calibration, shows a clear energy dependence (figure 5(b)). *χ* rapidly increases in value between 70 and 125 MeV before plateauing at a value of ∼ 205 MU Gy^−1^. No clear dependence on nozzle current is seen. Fitting equation (5) to this dataset, with the parameters given in table 1, results in a R^2^ of 0.999. The Δ parameter (equation (6)), representing the dose independent component, has a more complex relation to energy and cyclotron current. Overall, the parameter decreases with increasing cyclotron current (figure 5(c)). However, the rate of this decrease is energy dependent; lower energies showing a much steeper gradient which reduces quickly as energy increases before plateauing at ∼150 MeV. Equation (6) is fitted to this data as a surface with the parameters given in table 1, resulting in an R^2^ of 0.970.

**Table 1.** The fit parameters used for dosimetry equations (4)–(7).

Parameter	Value
${x}_{1}$	4.615e-02
${x}_{2}$	6.913
${x}_{3}$	2.056e+02
${d}_{1}$	−3.699e-02
${d}_{2}$	5.213
${d}_{3}$	−5.389
${t}_{1}$	3.613e-12
${t}_{2}$	4.086
${t}_{3}$	1.235e-13
${t}_{4}$	1.094e-01
$a$	2.429e-02
$b$	−6.587e-04
$c$	9.689E-07
$d$	−2.426e-02
$e$	8.322e-07
$f$	−3.347e-05
g	5.5290e-07
h	−2.255e-04
i	1.023

**Figure 6.**
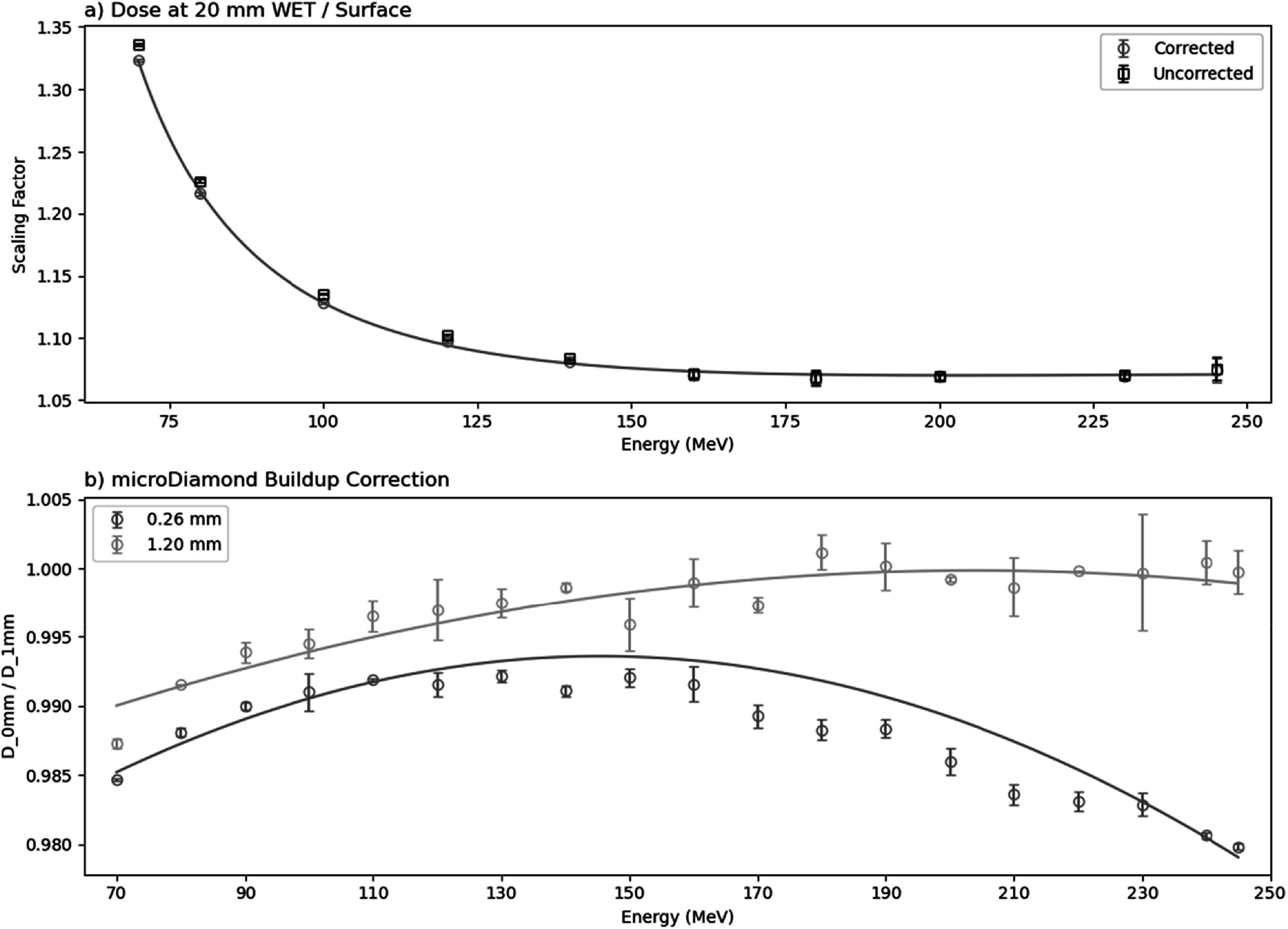
(a) The measured dose at 20 mm WET relative to ‘surface’ dose with matched deliveries as a function of energy with the fit from equation (7) shown as the line. The measured dose ratio is shown with correction for the 1 mm WET microDiamond buildup material with 1.2 mm polystyrene representing the modified 96-well plate. (b) Simulated dose measured distal to 0.26 and 1.2 mm of polystyrene (cell layer) divided by the same dose with an additional 1 mm of water (the microDiamond effective point of measurement). Lines shows a second order polynomial fit. Relying on microDiamond dose for the sample, without correction, results in an overdosing of ∼1%–2% for 0.26 mm polystyrene and ∼0.2%–1.0% for 1.2 mm polystyrene.

The *χ* and Δ terms combine to give the dose per MU calibration factor (equation (4)), measured at 20 mm WET (figure 5(d)). A clear low-dose non-linearity can be seen in the calibration factor. The rate of change in calibration factor as dose decreases correlates with achievable nozzle current, with higher currents resulting in steeper gradients in the low dose region. This is highlighted in figure 5(d), showing the impact of nozzle current on the calibration factor for multiple currents at 100 and 245 MeV. At higher doses the calibration factor tends towards a constant value, with lower energies settling at higher calibration factors for the same dose. Incorporating the equations (5) and (6) into equation (4), giving the fits shown in figures 6(d) & (e), gives an overall average R^2^ of 0.959.

The dose predicted by equation (4) (figures 5(d) & (e)) is corrected from the 20 mm WET reference depth to the ‘surface’ position for biological irradiations (equation (7)), shown in figure 6(a). This scaling is largest at lower energies where the increased Bragg peak dose gradient is apparent at the 20 mm WET reference depth, with a value of 1.32 for 70 MeV. This reduces as energy increases, reaching 1.07 at 245 MeV. The correction for the 1 mm WET microDiamond buildup material is shown in figure 6(b), for the 1.2 mm polystyrene used in these measurements and for another typical cell culture plasticware of 0.26 mm thickness. As the polystyrene thickness increases the correction plateaus for increasing energy, where the proton dose buildup completes in the plastic. Not accounting for this correction results in an underdosing for energies below ∼160 MeV, with ∼1.3% underdosing at 70 MeV.

**Figure 7.**
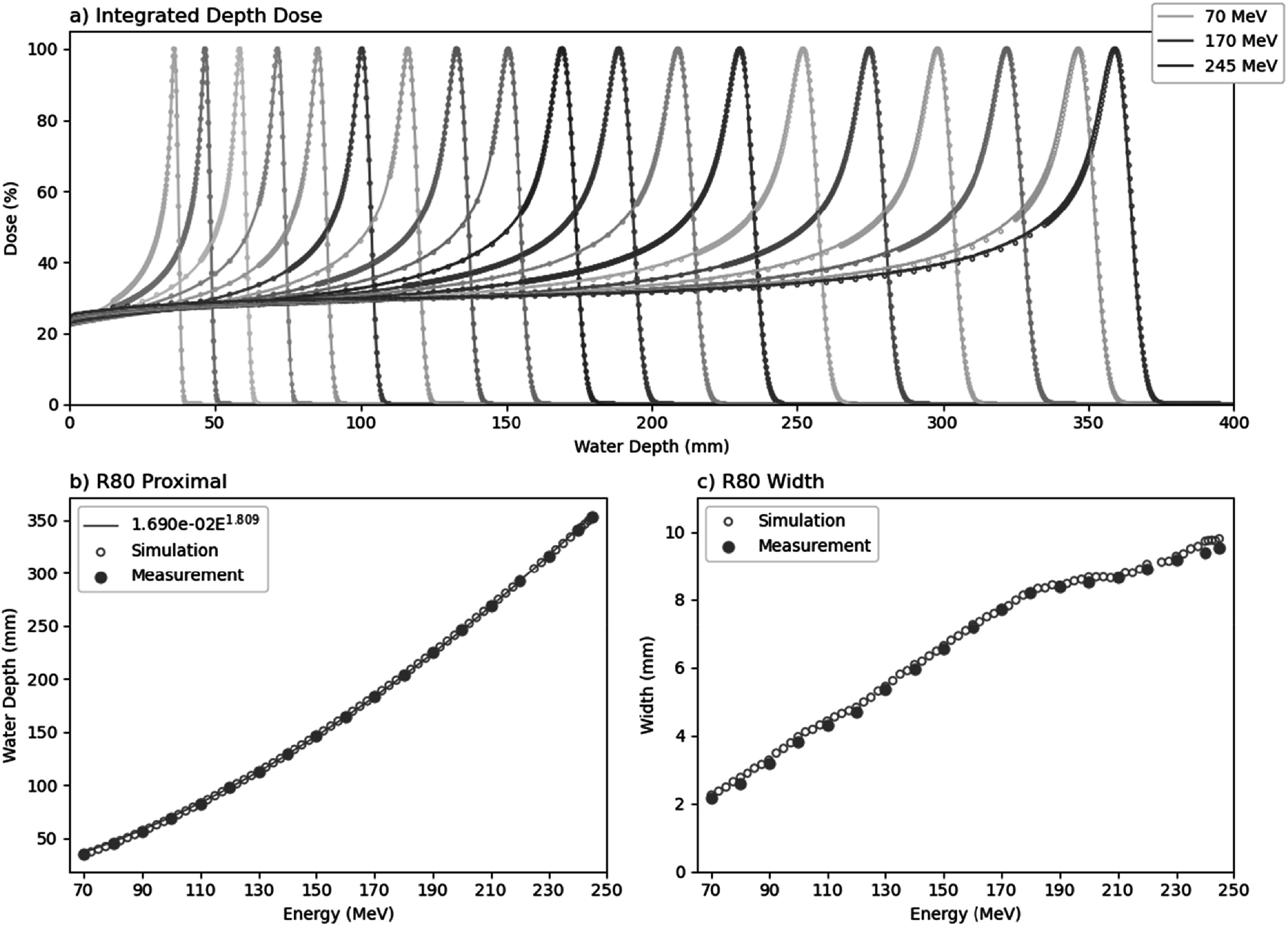
(a) The integrated depth dose measured in water between 70 MeV to 245 MeV at 10 MeV intervals. Values are self-normalised to the maximum dose per energy. Points show measurement and lines show simulation (b) The interpolated range of 80% dose maximum proximal to the Bragg peak. Filled points show measurement, empty points show simulation, and line shows Bortfeld fit (c) The width of the Bragg peak between distal and proximal depths of 80% dose maximum. Filled points show measurement and empty points show simulation.

The fit parameters of equations (4)–(7), shown in figures 6 and 7, are given in table 1.

### Beam energies

The integrated depth dose (IDD) was measured with the PTW MP3-M watertank using a PTW thin window chamber and PTW Bragg peak chamber (figure 7). The WET of the PTW thin window chamber and the watertank window were separately measured with a PTW PeakFinder at 230, 150, and 70 MeV giving 2.029 ± 0.102 mm and 5.952 ± 0.088 mm respectively. IDDs were measured at 10 MeV intervals between 70–245 MeV, shown in figure 7(a). The range in water of the proximal 80% maximum dose follows the power law in equation (8), with parameters a = 1.690e-02 and p = 1.809 (figure 7(b)). The width of the Bragg peak, given as the distance between proximal and distal 80% maximum dose, is shown in figure 7(c).

There is good agreement between measured dose depth and the TOPAS beam model (figure 7). However, whilst the model reproduces the majority of the range (figures 7(a) and (b)) it struggles to fully capture the fall off past the Bragg peak (figure 7(c)). The model shows a slightly wider peak than was measured across the energy range.

**Figure 8.**
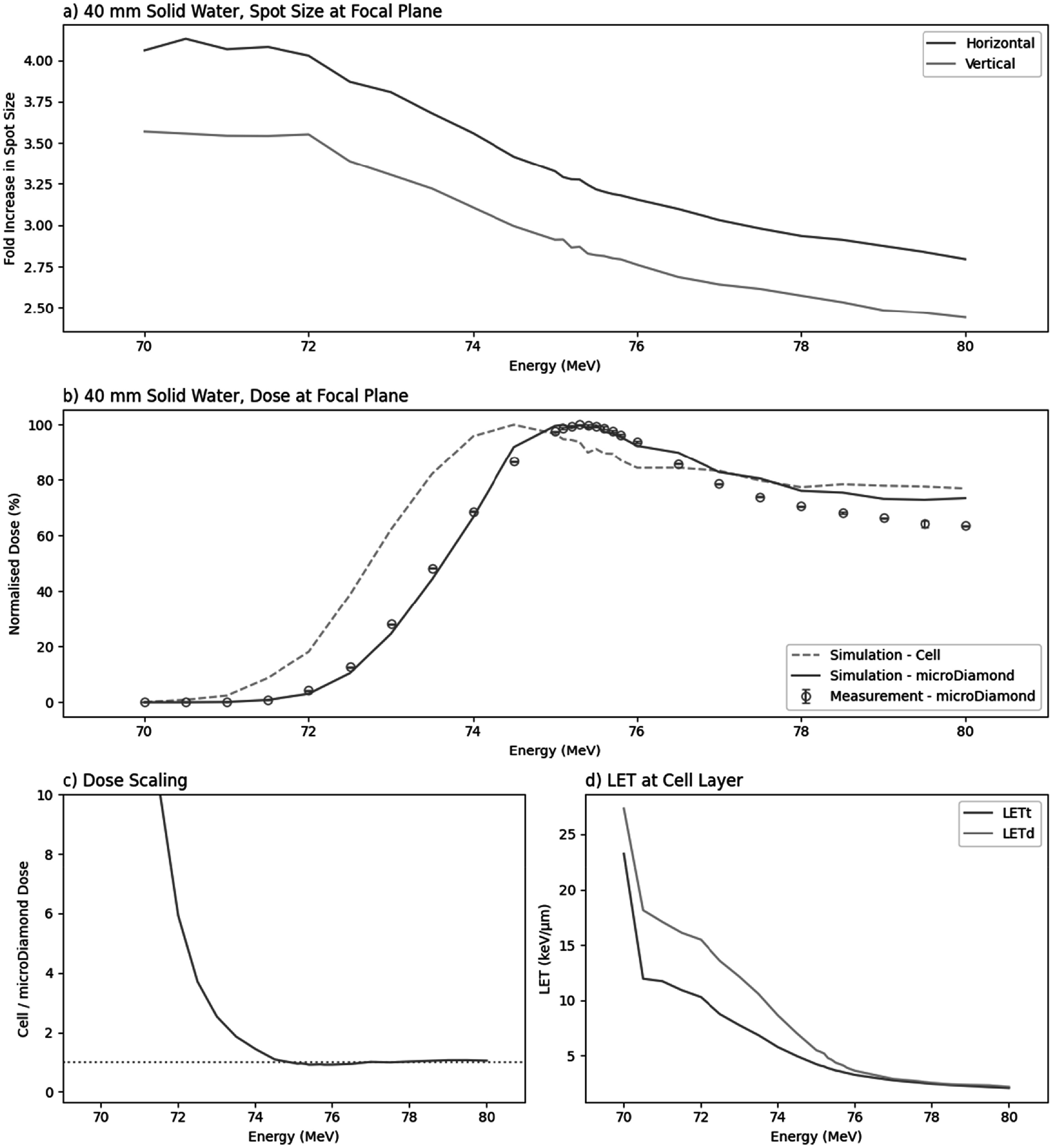
The effect at the focal plane of extra degrading with 40 mm solid water attached to the nozzle. In all cases, energy refers to nominal energy from the energy selection system. (a) The simulated fold increase in spot size at the focal plane. (b) The normalised dose measured (points) at the focal plane by PTW microDiamond, line shows simulation through TOPAS, dashed line shows the simulated equivalent dose at the cell layer. Error bars show standard deviation between three repeated measurements (smaller than the marker size). (c) The simulated ratio of cell dose to microDiamond dose, used to scale measured dose for biological irradiation. The dotted line shows a scaling of 1, where dose to the cell layer is equal to microDiamond dose (d) The simulated track- and dose-averaged LET at the cell layer.

High-throughput biological irradiation with energies lower than 70 MeV is achieved by extra degradation with 40 mm of solid water attached to the nozzle. The 640 mm air gap between the solid water and biological sample causes a dramatic increase in the spot size as predicted by the TOPAS beam model (figure 8(a)), significantly reducing dose density and increasing required delivery time. For all energies investigated, up to 80 MeV, the solid water results in at least a 2.5x increase in spot size at the focal plane.

Dosimetry of the degraded beam was measured *in situ* with the PTW microDiamond at the focal in the energy range 70–80 MeV. The measurement was in the TOPAS beam model simulation with and without the 1 mm WET microDiamond buildup, scoring dose to the ‘cell layer’ (figure 8(b)). Here, we see a shift in maximum dose to the cell layer at lower energy due to the extra 1 mm range. The ratio of simulated doses, with and without the microDiamond gives a scaling factor of *in situ* measured dose and dose delivered to cells (figure 8(c)). This ratio settles to ∼1 after 76 MeV and rapidly increases as energy decreases. The LET, dose-averaged (LET_d_) and track-averaged (LET_t_), achieved at the cell layer following 40 mm of solid water is shown in figure 8(d).

### Dose rate

**Figure 9.**
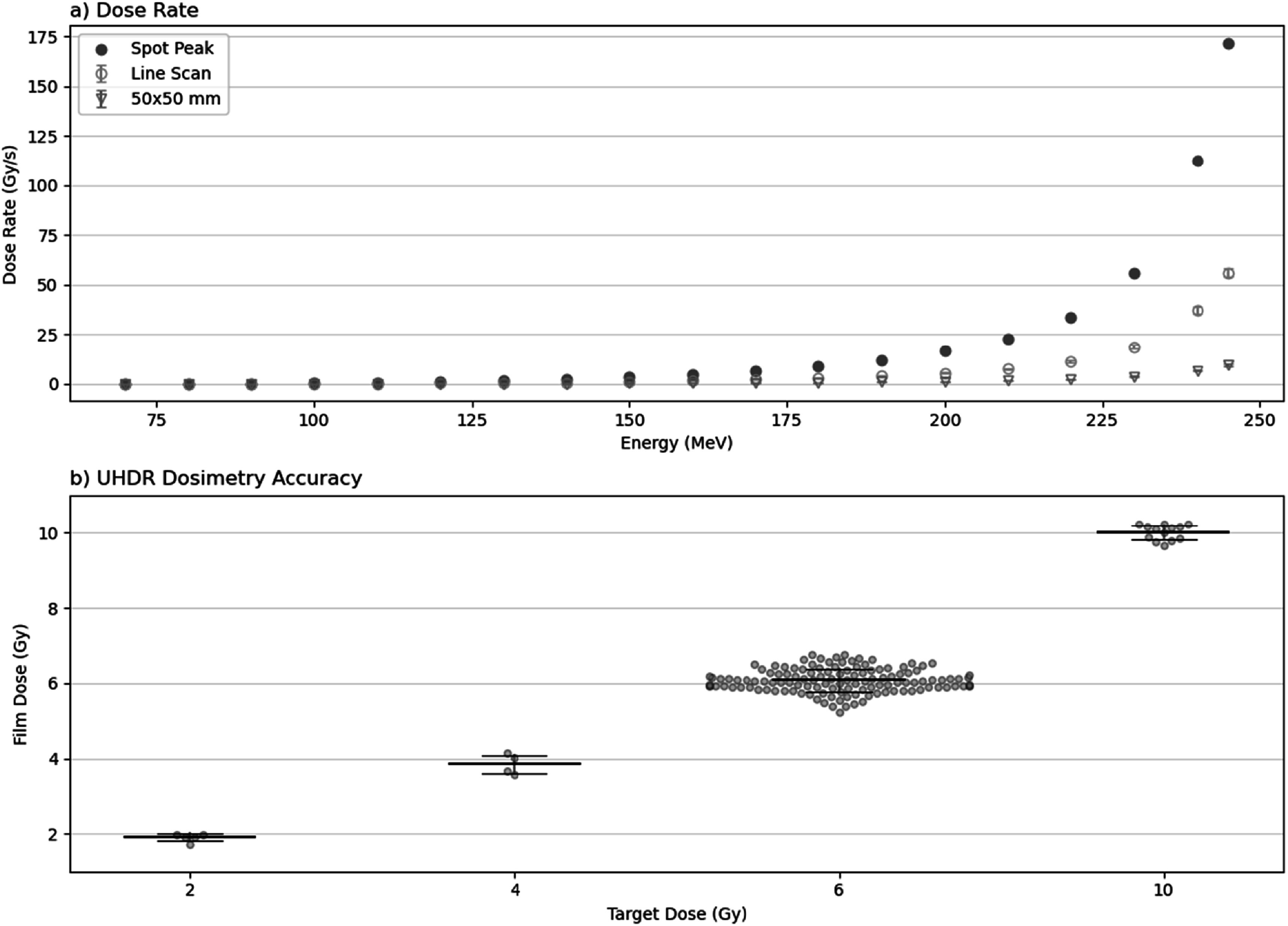
(a) The calculated peak dose rate of the 2D Gaussian distribution for a single spot at 800 nA cyclotron current as a function of energy based on measurements for 245 MeV (filled symbols). The average dose rates for a single horizontal line scan (150 mm, 2.5 mm spot spacing) of 6 Gy delivery as a function of energy (open circle symbols). The average dose rate for a T25 flask irradiation (50 × 50 mm, 2.5 mm spot spacing) is shown as open triangles. (b) The measured film dose for UHDR irradiated *in vitro* samples as a function of target dose, symbols show individual film measurements, lines show mean and one standard deviation.

Measurement of single spot dose rate for a 245 MeV beam gave a value of 186.32 Gy s^−1^ after accounting for cyclotron current fluctuations, transmission, and the 3.8 mm^2^ microDiamond active area and Gaussian spot profile measured on the day. Figure 9(a) shows the spot peak dose rate achievable with current focusing performance for all energies, calculated by scaling the reference dose rate above with transmission (figure 5(a)) and relative spot size (figure 3(a)). Current maximum spot peak dose rate is achieved at 245 MeV, reaching 171.93 Gy s^−1^. This falls off rapidly with energy, dropping to 112.65 Gy s^−1^ in just 5 MeV at 240 MeV, and reducing to just 0.05 Gy s^−1^ at 70 MeV.

UHDR MUs are tuned with the microDiamond prior to delivery with dose later verified through film analysis, which is shown in figure 9(b). Mean film dose with an uncertainty of one standard deviation was 1.91 ± 0.09, 3.85 ± 0.24, 6.08 ± 0.31, and 10.01 ± 0.20 Gy for target doses of 2, 4, 6, and 10 Gy respectively.

Operating at the maximum energy, 250 MeV, will increase transmission from the current maximum of 7.8% for 245 MeV. Extrapolating the fit to transmission (figure 5(a)) to 250 MeV suggests a transmission of 11.5%. This would result in an achievable dose rate for a 50 × 50 mm target of 14.1 ± 0.6 Gy s^−1^ for 6 Gy, or 87 ± 5 Gy s^−1^ for a line scan of 6 Gy.

## Discussion

The radiobiology experimental endstation (figure 2) is a world’s first that can provide tight environmental control during proton irradiation, minimising unaccounted for confounding factors that will lead to a reduction in experimental uncertainties. The integrated robotics system provides a high-throughput platform to maximise research time and allows for *in situ* quality assurance, minimising uncertainties arising from radiation delivery. The recent upgrades, chiller unit and fluorescent microscopy, will further the research capabilities and open new areas of study for the community.

The beam optics result in a relatively non-divergent beam through the nozzle (figure 3(b)), allowing for simple planning of SOBP or irradiations at depth in water. This also provides a small robustness against depth alignment to the focal plane, where, due to the non-divergent beam, we are less sensitive to changes in dose flux. The spot sizes across the energy range (figure 3(a)) are relatively large compared to adjacent clinical gantries. For example, at 245 MeV we achieve spot sizes at the focal plane to one standard deviation of 4.4 x 5.7 mm. Clinically, the equivalent spot size is ∼3 × 3 mm. For most *in vitro* irradiation this larger spot size is acceptable, where deliveries tend to use uniform fields. This mainly has implications on dose rate (figure 9(a)), which reduces achievable dose rates and results in longer delivery times for low energy irradiation. Given the clinically achieved spot size, some work remains to further optimise beam optics. This will also alleviate any beam clipping on inline diagnostic equipment in the research line and could increase transmission. Particularly at pinch points such as the beam profile monitors, where we currently have a restriction in the vacuum chambers. Similarly, the DKW physical air gap acts as a scattering source resulting in an increase in the beam profile exacerbating clipping issues at pinch points. Spot positioning is repeatable (figure S1), and we see no improvement with lower tolerances or detriment in irradiation time, implying power supplies are well below the tolerance applied. This level of repeatability is similar to other facilities (Li *et al* 2013), and is unlikely to impact field dose homogeneity (Arjunan *et al* 2021). The homogeneity index is within our 3% tolerance threshold across the energy range (figure 4(g)). There appears to be no energy dependent trend, implying good dose and scanning control across the range. The effect of increased spot size is apparent as a reduction in area of the homogenous region (figures 4(a)–(c)). Further optimising spot size, particularly at low energy, will reduce the required overscan area for *in vitro* samples necessary to provide uniform irradiation.

The dosimetry equation (equation (4)) has both a dose dependent, *χ*, and dose independent, Δ, component in the denominator which determines its behaviour. *χ* depends only on energy and serves to translate the calibration factor linearly. Δ depends on both the energy and the transmitted beam current, which ultimately depends on the cyclotron current. Δ determines the rate of change of the gradient in calibration factor against target dose at lower doses, with higher Δ values resulting in a faster change in gradient and therefore larger changes in calibration factor for small changes in target dose. The dominant factor influencing the value of Δ is the nozzle current, due to the IC 101’s discrete sampling periods and deadtime, with energy playing a smaller role. A shift in cyclotron current away from the expected and requested current, which can sometimes exceed 10%, can have substantial implications on dose accuracy. This is particularly problematic for SOBP delivery, where low energy peaks tend to have low dose contributions at the 20 mm WET reference position. This sensitivity can be reduced by adopting an energy specific spot spacing. Fields of dose are delivered by specifying the dose to be delivered to each spot in a spot map. By decreasing spot density, the required contribution of dose from each spot to maintain the same field dose increases, moving towards a domain in equation (4) where the Δ parameter, and therefore cyclotron current, has less influence (i.e. moving to higher doses in figure 5(d)). Currently all deliveries use 2.5 mm spot spacing, which is smaller than our smallest spots of 4.4 mm at 245 MeV. Acceptable homogeneity should still be achievable with spacings of up to 5/3 spot sigma, allowing us to increase spot spacing up to 7.3 mm for 245 MeV and 17.8 mm for 70 MeV. Equation (4) is routinely verified as part of monthly QA for the test cases of 70 MeV at 800 nA cyclotron current and 230 MeV at 56 and 28 nA cyclotron currents, with doses in the range of 0.25–2 Gy. Typically, we see dose inaccuracies on the order of ±1%–2%. Poorer repeatability is seen for 70 MeV, which could be the result of alignment due to an increased sensitivity to 20 mm WET positioning. Dose repeatability between sequential fields is on the order of <0.1%.

*In situ* Dosimetry can be quickly verified at the beginning of an experimental shift by point measurements the dose of a reference field with the PTW microDiamond. The discrepancy between ‘surface’ dose that cells would be exposed to and the measured dose on the PTW microDiamond (figure 6(b)) depends on the energy and thickness of cell culture plasticware. For thicker plasticware the discrepancy is reduced at higher energy, where charged particle equilibrium is achieved in the plastic, and increased at lower energy, where the plastic shifts the cell layer into a deeper position and steeper dose gradients from the Bragg peak have a larger effect. Figure 6(a) shows an increasing scaling factor at energies greater than approximately 180 MeV, with values of 1.068 and 1.075 at 180 and 245 MeV respectively, due to nuclear buildup effects, which is not corrected for. Relying on microDiamond dose alone, without correction of the 1 mm WET microDiamond buildup, is likely to cause an under dosing. This isn’t corrected for in our standard practice. Accounting for this effect is possible and may be necessary, especially when comparing the biological response between x-ray and protons where a small effect is expected. For example, measuring a 10% relative change in cell survival could be masked by the combination of proton dosimetric uncertainties (equation (4), figure 6, focal plane depth offsets, angular shift) and x-ray dose uncertainty (buildup effects, homogeneity of delivery, dosimetric calibration).

The disagreement between the simulated and measured Bragg peak widths (figure 7(c)) is likely due to an assumed normally distributed initial energy in the simulation. The energy distribution is likely to be skewed with a slight low energy tail due to some beam clipping at vacuum chamber restriction sites. This is being investigated through a complete beamline simulation. The effect is manifested when there are increased sensitivities to range, such as the increased energy degradation setup (figure 8). With this uncertainty in relative position, significant underdosing can occur. This is difficult to measure *in situ* with an increased uncertainty in film dose at increased LET (Gambarini *et al* 2019, Guan *et al* 2023). In the meantime, SOBPs are now routinely used for high-LET irradiations. This reduces range uncertainty, giving more confidence in delivered dose at the cost of uncertainty in LET and an increased spectrum of LET on the sample. The distance between solid water degraders and the sample leads to a significant increase in spot size (figure 8(a)). This decreases dose density and increases the irradiation time. This method was necessary to free up space in the radiobiology endstation. Alternative approaches, such as local degradation with higher Z material attached to an *in vitro* sample, are being investigated.

The achievable dose rate for UHDR irradiation depends heavily on the field geometry and therefore sample type (figure 9(a)). To maximize dose rate, we only use 245 MeV beams, which achieves both maximum transmission (figure 5(a)) and minimum spot size (figure 3(a)). Using this we can achieve dose rates of 9.8 + 0.5 Gy s^−1^ for 6 Gy on a 50 × 50 target area required to cover a T25 flask that may be used for clonogenic assays. To further enhance the dose rate, we can deliver a single horizontal line across the sample, reaching 56.0 ± 3.0 Gy s^−1^ for 6 Gy. This requires biological samples with adherent cells and to be kept in a well-known orientation throughout irradiation and analysis. The most significant gain in transmission can be achieved by increasing energy to 250 MeV, potentially increasing from 7.8% at 245 MeV to 11.5% at 250 MeV. Improved focussing through quadrupole optimisation and removal of the dual-Kapton window will increase dose density and reduce spot-spot influence over a scan area, further increasing delivered dose rate. This would result in an achievable dose rate for a 50 × 50 mm target of 42 ± 4 Gy s^−1^ for 6 Gy, or 420 ± 20 Gy s^−1^ for a line scan of 6 Gy, although this does come at a considerable burden on the required positional accuracy as the spot size is significantly reduced.

## Conclusions

This work has provided an overview of the performance and capabilities of the proton beam therapy research facility at the University of Manchester and Christie NHS foundation trust, framed around medical physics capabilities. In summary, samples can be irradiated with protons in the energy range of 70–245 MeV. The biological effect of end of range protons (increased LET) can be investigated by further degrading the beam energy in the room—effectively down to 0 MeV. The proton spot can be scanned across an area of 400 × 300 mm, with dose homogeneity less than 3%. Dosimetric variation between repeated fields is typically <0.1%. Samples can be irradiated at UHDR with 245 MeV at a maximum dose rate of 171.92 Gy s^−1^ in the centre of a single spot, or at a maximum average dose rate of 56 ± 3 Gy s^−1^ for a single horizontal line scan. Whilst these capabilities are likely to be similar to other Varian facilities, we have designed the workflows and irradiation schemes with *in vitro* biology in mind. Our bespoke research beamline provides a level of control that is not readily available at many clinical facilities, giving the versatility required to achieve experimental aims. For example, addition of collimators into the beamline for spatially fractionated radiotherapy is possible. Given the required level of versatility, and the unpredictableness of experimental needs, we have largely presented the results of physics capabilities. With this provided information, an experimentalist can assess the feasibility of carrying out their research at our facility.

## Data Availability

All data that support the findings of this study are included within the article (and any supplementary files).
